# Odor-Induced Vomiting Is Combinatorially Triggered by Palp Olfactory Receptor Neurons That Project to the Lobus Glomerulatus in Locust Brain

**DOI:** 10.3389/fphys.2022.855522

**Published:** 2022-04-20

**Authors:** Liyuan Sun, Xueqin Pan, Hongwei Li, Xinyang Zhang, Xincheng Zhao, Liwei Zhang, Long Zhang

**Affiliations:** ^1^ Department of Entomology, China Agricultural University, Beijing, China; ^2^ Institute of Plant Inspection and Quarantine, Chinese Academy of Inspection and Quarantine, Beijing, China; ^3^ Department of Zoology, University of Cambridge, Cambridge, United Kingdom; ^4^ Department of Entomology, Henan Agricultural University, Zhengzhou, China; ^5^ Shandong Academy of Agricultural Sciences, Jinan, China

**Keywords:** vomiting, odor, odorant receptor, palp sensilla, olfactory receptor neuron, lobus glomerulatus

## Abstract

Although vomiting is commonly recognized as a protective reaction in response to toxic stimuli, the elaborate sensory processes and necessary molecular components are not fully clear, which is due to a lack of appropriate experimental animal models. Vomiting reflex to volatile chemicals renders locust one candidate for vomiting model. Here, we identified a panel of chemical cues that evoked evident vomiting in locust nymphs and demonstrated the selected combinatorial coding strategy that palps but not antennae olfactory receptor neurons (ORNs) employed. Specifically, knocking down individual palp odorant receptors (ORs) such as OR17, OR21, and OR22 attenuated the vomiting intensity evoked by E-2-hexenal and hexanal, while suppression of OR12 and OR22 augmented vomiting to E-2-hexenal and 2-hexanone, respectively. Furthermore, dual-RNAi treatment against OR17 or OR21 together with OR22 resulted in a much lower response intensity than that of individual OR suppression. Furthermore, OR12 was revealed in palp sensilla basiconica (pb) subtype 3 to tune the neuronal decaying activity to E-2-hexenal. Finally, anterograde labeling indicated that palp ORNs primarily projected into the lobus glomerulatus (LG), and the projection neurons (PNs) in the LG further projected into the accessary calyx (ACA). Together, the establishment of an olfaction-inducible vomiting model in locusts deepens the understanding of olfactory coding logics and provides an opportunity to clarify the neural basis underlying animal vomiting.

## Introduction

Vomiting is a commonly unpleasant reaction of visceral discomfort that indicates potential sickness or poisonous compounds. Vomiting is characterized by the discontinuous ejection of the contents from the stomach, usually in a series of involuntary spasmic movements. There are various associated stimuli that evoke vomiting, including toxins, pathogenic fungi/bacteria, irritant chemicals, motion sickness, and medical treatments, such as chemotherapy (Andrews, 1992; Miller and Leslie, 1994; Hornby, 2001). Despite its extensive occurrence and associated potential health risks, our understanding of the molecular and cellular pathways capable of causing vomiting remains limited. One of the major obstacles restricting our understanding of how vomiting is caused and controlled is the lack of suitable animal models, especially genetically editable models. Unfortunately, commonly used mammalian model mice lack a significant vomiting response (Andrews, 1992; Andrews and Horn, 2006; Horn et al., 2013), even though nausea, which is frequently associated with vomiting, has recently been molecularly and morphologically dissected in mice (Zhang et al., 2020).

In addition to humans, many invertebrates, such as insects, vomit in response to physical and chemical stimuli. The widespread genetic conservation between vertebrates and invertebrates might provide a chance to gain fundamental insights into vomiting. Most insects have developed innate avoidance behaviors, similar to nausea in mice, to get rid of toxins. For example, the fruit fly, *Drosophila melanogaster,* responds negatively to the CO_2_ released by stressed conspecifics (Suh et al., 2004; Faucher et al., 2006), as well as to the toxic fungal product geosmin (Stensmyr et al., 2012). However, the fruit fly is incapable of vomiting. Many orthopteran locusts vomit in response to external stimulation, including forceful physical touch and high doses of chemicals. The vomit of locusts consists of partially digested food plus a digestive enzymatic mix that is toxic or deadly to predators (Freeman, 1967, 1968; Eisner, 1970; Capinera and Sechrist, 1982). Potential benefits of vomiting include threatening predators or activating protective systems to remove unpleasant cues. In addition, the versatile genetic manipulation approaches using RNAi (Wynant et al., 2012; Zhang et al., 2017) and CRISPR–Cas9 (Li et al., 2016; Guo et al., 2020) have been demonstrated to be highly effective and easily applied in locusts, thus providing powerful ways to investigate how vomiting occurs and is controlled. Collectively, locusts are notably the ideal model to study vomiting.

The key stimuli and underlying sensory basis on vomiting have remained enigmatic. Two critical stimuli in locusts are physical touch, namely, mechanical force and olfactory cues, namely, odorants. A locust can vomit when we squeeze its head or abdomen. A previous study indicated that one odorant, E-2-hexenal, is capable of causing locusts to vomit (Zhang et al., 2017). Consistent with vertebrates, volatile irritants are implicated in the vomiting response, but a panel of active odorants and the corresponding olfactory sensors are not clearly clarified. The canonical olfactory receptor complex contains two components: odor-specific odorant receptors (ORs) and an ubiquitous odorant coreceptor (Orco). Ionotropic receptors (IRs) act as another olfactory receptor family, reacting to organic acids and amines (Abuin et al., 2011). At present, the olfactory receptors and potential neural circuits involved in locust vomiting remain largely unexplored. Identifying the diversity of the causal odorants and molecular components that induce vomiting could deepen the understanding of how vomiting occurs.

In this study, we systematically examined the effect of a large panel of volatiles on inducing locust vomiting and also performed reverse genetic screening of multiple ORs expressed on the olfactory organ, palps. A combinatorial coding network of diverse active odorants and multiple olfactory receptors was found in the modulation of vomiting response. This behavior primarily relies on olfactory receptor neurons (ORNs) in palp sensilla basiconica to receive the odorants and to transmit the information into the lobus glomerulatus (LG), and the projection neurons (PNs) in the LG projected into a higher brain area, the accessary calyx (ACA). Taken together, this evidence lays the foundation for developing locusts into a novel vomiting model that is triggered by volatile compounds.

## Materials and Methods

### Animals

Locusts (*L. migratoria*) were obtained from the Department of Entomology, China Agricultural University. They were reared in crowded conditions (28–30°C, 60% relative humidity, and 12:12 h light:dark cycles) and fed daily with fresh wheat shoots. Three- to five-day-old 5^th^ instar nymphs were used for experiments.

### Chemicals and Preparation

All chemicals were ordered with the highest purity. Working solutions were prepared with paraffin oil. The chemical compounds for vomiting behavior experiments and single sensillum recordings are listed in Supplementary Tables S4 and S5, respectively.

### Vomiting Experiment

Starved locusts (12 h) with antenna ablation or both antenna and palp ablation were restrained within 1.5 ml Eppendorf tubes, leaving their heads and palps free to move. All the tubes were immobilized on a rack, and each tube was covered with a 30 mm diameter lid to form an independent confined space. Before each stimulation, only the lid of the tube containing the locust to be tested was opened, and the others were kept closed. Experiments were performed in a warm environment with a temperature of 28–30°C. Before each experiment, all animals were warmed in the test area for at least 10 min for adaptation. Each experimental group contained 24 locusts. 10 µl of the diluted compound was applied to a small piece of filter paper (2 cm × 0.5 cm, L×W) inserted into a Pasteur pipette. The pipette was changed every eight locusts, so three Pasteur pipettes were needed for each compound. Paraffin oil was used as a blank control. The pipette’s opening was placed 1 cm from the locust’s antenna/mouthpart. Chemicals on the filter paper were wafted to the mouthpart by a stimulus air controller (CS-05, Syntech, Netherlands). The airflow rate of the controller was set to 20 ml/min for a stimulation period of 1 s. There was a 10 s interval between the two testing insects. All vomit individuals were counted from each group. Each experimental group has only one replicate, and per treatment, at least three independent groups are needed. The vomiting ratio was defined as the number of vomiting individuals divided by the total number of insects tested.

### *In Situ* Hybridization on Sections and Whole-Mount Fluorescence *In Situ* Hybridization

Digoxigenin (DIG)-labeled antisense and sense probes were generated as described by Xu et al. (2017). Templates of ORs were prepared by gene amplification (Supplementary Table S1) and cloned into the pGEM-T vector, and probes were transcribed from linearized recombinant pGEM-T plasmids using the T7/SP6 RNA transcription system (Roche, Basel, Switzerland). Single ISH on tissue sections was performed as described earlier (Xu et al., 2017; Zhang et al., 2017). WM-FISH was performed with freshly prepared maxillary palps, which were dissected from cold anesthetized animals. A hole was cut near the dome of the palp with a freezing microtome. This treatment made it easier for solutions to reach target tissues. The palps were then directly transferred to the fixation solution [4% paraformaldehyde in 0.1 M sodium bicarbonate (NaHCO_3_), pH 9.5] and incubated overnight (12–14 h) at 4°C. All incubations and washes were performed in thin-walled PCR tubes (0.25 ml; Axygen, United States) with slow rotation or moderate shaking. After fixation, palps were washed for 1 min in PBS (phosphate-buffered saline = 145 mM NaCl, 1.4 mM KH_2_PO_4_, 8 mM Na_2_HPO_4_, pH 7.1) with 0.03% Triton X-100; incubated for 10 min in 0.2 M HCl with 0.03% Triton X-100; and washed for 2 min in PBS with 1% Triton X-100. Subsequently, palps were prehybridized at 55°C for at least 6 h with *in situ* hybridization solution (50% formamide, 5x SSC, 1x Denhardt’s reagent, 50 μg/ml yeast RNA, 1% Tween 20, 0.1% Chaps, and 5 mM EDTA pH 8.0). After prehybridization, palps were incubated in a hybridization solution containing labeled antisense RNA probes at 55°C for at least 90 h. In control experiments, labeled sense RNA probes instead of antisense RNA probes were used. After hybridization, the antennae were washed four times for 15 min each in 0.1xSSC (1xSSC = 150 mM NaCl, 15 mM Na-citrate, pH 7.0) and 0.03% Triton X-100 at 60°C and then treated with 1% blocking reagent (Roche) in TBS (100 mM Tris, 150 mM NaCl, pH 7.5) with 0.03% Triton X-100 for at least 5 h at 4°C. DIG-labeled RNA probes were detected by using an anti-DIG AP-conjugated antibody (Roche) diluted 1:250 in TBS, 0.03% Triton X-100, and 1% blocking reagent. After at least 90 h at 6°C, palps were washed five times in TBS with 0.05% Tween 20 for 10 min each. DIG-labeled probes were visualized by incubation in the dark for 6 h with HNPP (2-hydroxy3-naphthoic acid-2′-phenylanilide phosphate, Roche) at a concentration of 1:100 in DAP-buffer (100 mM Tris, 100 mM NaCl, 50 mM MgCl_2_, pH 8.0) at 6°C. Finally, palps were washed in TBS with 0.05% Tween 20 three times for 5 min each and briefly rinsed in PBS before they were mounted in PBS/glycerol (1:3).

### RNA Interference

Double-stranded RNA (dsRNA) was synthesized based on the manufacturer’s manual. In brief, PCR products were amplified with T7 promoter-conjugated primers (Supplementary Table S2) and then purified with Wizard® SV Gel and PCR Clean-Up System (Promega, United States) as templates for *in vitro* transcription. DsRNA was synthesized with the T7 RiboMAX™ Express RNAi System (Promega, United States), and its concentration was determined with an ND-2000 spectrophotometer. DsRNA was then diluted to 2000 ng/μl with ddH_2_O and stored at −20°C. 5 µg of dsRNA was injected into each locust’s dorsal vessel through the abdomen’s intersegmental membrane (1st day of 5^th^ instar nymphs) by using an IM-9B microinjector (Narishige, Japan) equipped with a glass capillary. DsGFP was microinjected as a control group. The treated locusts were raised normally, similar to wild-type locusts. RNAi-treated animals were used for behavioral or electrophysiological experiments on the 3rd day post-injection. After these experiments, intact maxillary palps were isolated and immediately frozen in liquid nitrogen or kept at −80°C. Total RNAs were then extracted using TRIzol reagent. The silencing efficiency of RNAi was checked by RT–qPCR.

### Real-Time Quantitative PCR Analysis

Briefly, 1 µg of total RNA from RNAi treatments and control tissues was transcribed into cDNA (Promega, United States). RT–qPCR analysis was performed using two-fold diluted cDNAs, gene-specific primers (Supplementary Table S3), and a FastSYBR mixture (Tiangen, China) on an ABI QuantStudio 6 Flex Real-Time PCR system (Thermo Fisher Scientific). The actin gene was used as a reference. Relative expression levels of the target genes were calculated using the 2^−ΔΔCT^ method.

### Single-Sensillum Recordings

Single sensillum recordings were performed as described by Li et al. (2018b). The locust was restrained in a plastic tube, where the tip had been cut to allow passage of the head. Then, the assembly was fixed to a glass slide with tape and one of the maxillary palps was immobilized with thin tungsten on the prepared platform. Tungsten wire electrodes were sharpened electrolytically with 10% NaNO2. The recording electrode was inserted into the base of a basiconic sensillum through a motorized micromanipulator (CFT-8301D, C.M.D.T, China), and the reference electrode was inserted into the head. 10 µl of diluted odorant was placed on a small piece of filter paper (2 cm × 0.5 cm, L×W) and inserted into a Pasteur pipette. Stimuli were presented by placing the tip of the pipette through a hole in a glass tube carrying a constant stream (20 ml/s) directed at the locust and administering a 1 s pulse of charcoal-filtered air through the pipette containing the odorant. All stimuli were used for a maximum of three presentations. The recording electrode was connected to a 10× universal AC/DC amplifier (Syntech, the Netherlands). The recording signals were collected on an intelligent data acquisition controller (IDAC-4, Syntech, the Netherlands) and were calculated using Autospike32 (Syntech, the Netherlands).

### Staining of Palpal Sensory Neurons

Cold anesthetized locusts were immobilized with tape and dental wax in a Petri dish containing moist paper tissue. The dome region of the maxillary palp or the labial palp was cut, and crystals of the fluorescent dye, tetramethylrhodamine dextran (D7162, Invitrogen, Eugene, OR, United States) were applied at the cut surface by using a needle. The Petri dish was then placed in the dark at 4°C for 48 h for the diffusion of the dye. The brain and the SOG were subsequently dissected in insect saline and fixed with 4% paraformaldehyde at 4°C overnight before being rinsed in phosphate-buffered saline (PBS, pH 7.1) and dehydrated in an ascending ethanol series (50, 70, 90, 96%, 2 × 100%; 10 min each). Finally, the brain and the SOG were cleared in methylsalicylate and mounted in Permount.

### Staining of Olfactory Receptor Neurons in Single Sensillum

Cold anesthetized locusts were mounted on a slide. A glass piece was attached to the corner of the slide with double-sided tape to form a platform. The maxillary palp was aligned horizontally on the platform and fixed with dental wax. A single basiconic sensillum at the dome region of the maxillary palp was surrounded by a wall of wax. A droplet of distilled water was placed in the well so formed, and the sensillum was clipped off at its base with a broken glass microelectrode, exposing the sensory dendrites. Distilled water was replaced with a 10% (w/v) aqueous solution of micro-ruby. The animals were then kept in a dark and moist chamber at 4°C for 48 h. Subsequently, the brains were dissected, fixed, washed, dehydrated, cleared, and mounted in Permount as described earlier.

### Mass Staining

To stain all PNs in the LG, anterograde labeling was performed by applying dye into the LG. The sheath covering the LG was carefully removed, and crystals of Micro-Ruby attached to the tip of a microneedle were introduced into the LG. Dye-injected specimens were incubated in a dark and humid chamber for 4 h at room temperature. Subsequently, the brains were dissected, fixed, washed, dehydrated, cleared, and mounted in Permount as described above.

### Immunostaining for Identifying Neuropil Structures

To visualize the neural architecture in the central nervous system of the locust, the brain and the SOG were labeled by means of synapsin immunocytochemistry. After being fixed with 4% paraformaldehyde and rinsed in PBS, the preparations were preincubated with 5% normal goat serum in PBS containing 0.5% Triton X-100 (PBSX; 0.1 M, pH 7.4) for 3 h and then incubated with monoclonal antibody 3C11 (anti-SYNORF1, DSHB, United States) at a concentration of 1:100 in PBSX at 4°C for 5 days. After rinsing in PBS for 6 × 20 min, the preparations were incubated in a Cy2-conjugated anti-mouse secondary antibody (Invitrogen, Eugene, OR) at a concentration of 1:300 in PBSX at 4°C for 3 days. Finally, the preparations were rinsed for 6 × 20 min in PBS, dehydrated with ascending ethanol series, cleared in methylsalicylate, and mounted in Permount.

### Confocal Image Acquisition and Digital 3D Reconstruction

The fluorescently labeled samples were scanned with a confocal laser scanning microscope (TCS SP8, Leica, Germany) at 1024 × 1024 pixel resolution, a scanning speed of 100 Hz, a pinhole of size 1 airy, a line average of 4, and a step size between 2.5 and 3 μm. The brightness and contrast of the confocal images were adjusted in ImageJ. To visualize the three-dimensional structure of the brain and SOG, the confocal image stacks were subjected to reconstruction using Amira software (Amira 4.1, Visage Imaging, Fürth, Germany). The neuropil regions were reconstructed by using the segmentation editor, and the neuron axons were reconstructed by using the skeleton module.

### Statistical Analysis

GraphPad Prism 7 software was used to graph and statistically analyze data. All datasets were presented as mean ± SEM. We used two-way ANOVA with uncorrected Fisher’s LSD test, ordinary one-way ANOVA with uncorrected Fisher’s LSD test, or unpaired two-tailed t-test to analyze data from the vomiting behavior experiment, extracellular electrophysiology, and RT–qPCR (see indications in each figure legend). For all analyses, statistical notations are as follows: *p < 0.05; **p < 0.01; ***p < 0.001; and ****p < 0.0001.

## Results

### Palps Are Crucial Sensory Organs in Locust Olfactory Vomiting

**FIGURE 1:**
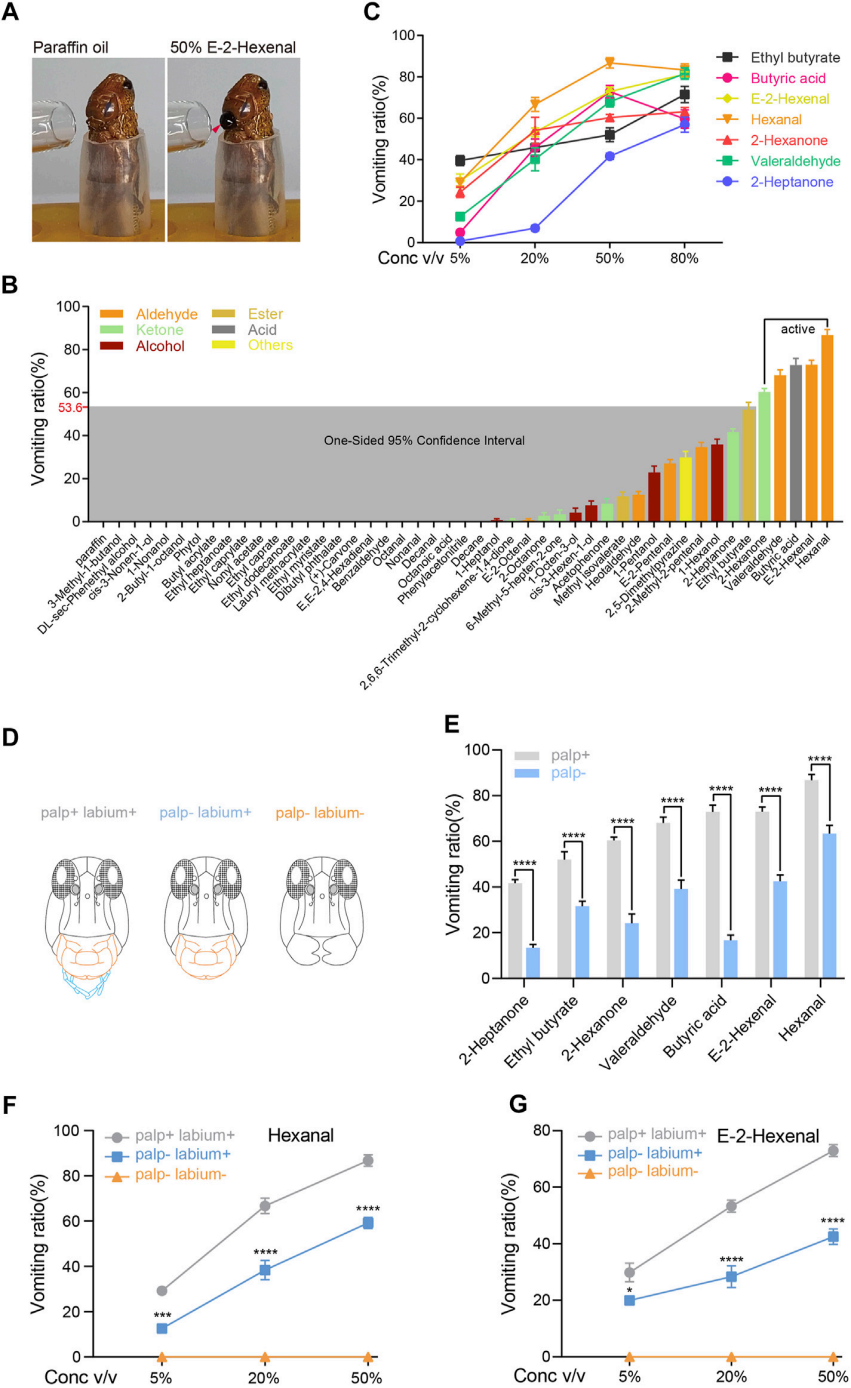
Palps are crucial sensory organs in locust olfactory vomiting. **(A)** The example of locust vomiting to 50% v/v E-2-hexenal and paraffin oil (solvent). The arrow indicates the vomit. **(B)** Vomiting ratio (the number of vomiting individuals divided by the total number of insects tested) of locusts to different kinds of odorants. All compounds were tested at 50% v/v dilutions in paraffin oil. *n* = 6 (24 locusts per replicate) for each compound. The shaded box marks the one-sided 95% CI of the mean vomiting ratio from all tested compounds. **(C)** The dose-dependent curve of locust vomiting in response to seven chemicals. *n* = 3–6 (24 locusts per replicate) for each independent test. **(D)** Frontal view of a locust head showing different treatments of the palp and labium. **(E)** Vomiting response assays of palp-retained locusts and palp-ablated locusts to seven chemicals. All chemicals were tested at 50% v/v dilution. n = 6 (24 locusts per replicate) for each assay. Statistical test: two-way ANOVA with uncorrected Fisher’s LSD test. **(F,G)** Vomiting response assays of locusts in different treatment groups in response to hexanal **(F)** and E-2-hexenal **(G)** at concentrations of 5–50% v/v. *n* = 6 (24 locusts per replicate) for each assay. Statistical test: two-way ANOVA with uncorrected Fisher’s LSD test. For all analyses, statistical differences are represented as follows: *p < 0.05, ***p < 0.001, and ****p < 0.0001. Data are represented as the mean ± SEM.

A previous study revealed that locusts with antenna ablation vomit to stimulation of 50% E-2-hexenal (v/v, in paraffin oil) at close proximity (Zhang et al., 2017) (Figure 1A). Interestingly, however, when we ablated both the maxillary and labial palps and stimulated the antennae at close proximity, none of the locusts vomited (Supplementary Figure S1A). This demonstrated that antennae are not required for odorant-induced vomiting. Next, to identify more volatile chemicals that evoke vomiting, a panel of 46 odorants derived from host and non-host plants, locust cuticular and fecal volatiles, were under investigation in behavior. These odorants represent a wide variety of chemical classes, including esters, alcohols, ketones, aldehydes, and acids (Supplementary Table S4). Moreover, the panel includes compounds with a broad range of chain lengths (4–20) and also odorants that may be particularly significant to locusts, such as phenylacetonitrile and 2,5-dimethylpyrazine.

Antenna-ablated animals were tested at 50% (v/v) dilutions, and the vomiting ratio was quantified as the percentage of vomiting individuals from the total number of locusts tested. Approximately 48% of tested compounds caused locust vomiting (Figure 1B). One compound is defined as “active” when its vomiting ratio reaches the upper limit of 53.6% which is calculated from a one-sided 95% confidence interval of the mean vomiting ratios from all tested compounds (Figure 1B). Ultimately, all five active compounds (hexanal, E-2-hexenal, butyric acid, aleraldehyde, and 2-hexanone), together with another two chemicals (ethyl butyrate and 2-heptanone) with a close vomiting ratio to the upper limit, were tested in the following experiments. Most selected compounds showed dose-dependent (from 5 to 50%) response in vomiting (Figure 1C). Together, locust vomits sensitively to a panel of active odorants in a dose-dependent manner, and this process is independent of antennae.

So far, we have proven that olfactory stimulation to locust mouthparts can induce vomiting. The maxillary and labial palps on the mouthpart might be the possible organs where olfactory coding occurs. The distribution pattern and microstructure of the palp olfactory sensilla have been intensively studied in locusts. To investigate whether olfactory inputs on palps contribute to locust vomiting, the palps were ablated before the vomiting assay (Figure 1D). As a consequence, palp-less locusts showed significantly reduced vomiting intensities in all seven tested compounds (Figure 1E). In addition, palp-dependent vomiting responses to hexanal and E-2-hexenal were attenuated under all three assayed doses (Figures 1F,G). Another mouthpart accessory organ, the labium, played an extremely critical role in evoking vomiting since the ablation of this organ completely removed all responses (Figures 1F,G). We also ablated the labium alone and found that none of the locusts vomited in response to the seven compounds used in Figure 1E (data not shown). In addition, semi-quantitative RT-PCR showed that the Orco gene was not expressed in the labium, but the IR8a and IR25a genes were expressed in the labium (Supplementary Figure S1B). These findings indicate that the labium may be directly required for vomiting.

### Odorant Receptors Are Involved in the Activation or Inhibition of Locust Vomiting

**FIGURE 2:**
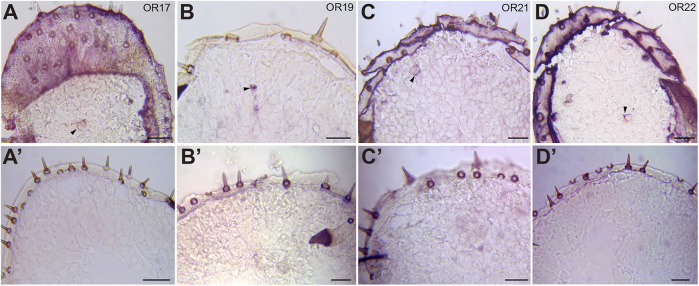
Expression of ORs in the maxillary palp of the migratory locust *L. migratoria*. *In situ* hybridizations were performed on sections of the maxillary palp with DIG-labeled antisense probes and sense probes. Signals were visualized using an anti-DIG antibody and color substrates. **(A–D)** ORNs expressing OR17 **(A)**, OR19 **(B)**, OR21 **(C),** and OR22 **(D)** were labeled. The black arrow indicates a cell expressing ORs. **(A′–D′)** No neurons were labeled by sense probes of OR17 **(A′)**, OR19 **(B’)**, OR21 **(C′),** and OR22 **(D′)**. Scale bars: 50 μm.

Two distinct olfactory pathways function on locust palps: one is mediated by OR-expressing neurons in the sensilla basiconica, and the other is processed by IR-positive neurons in the sensilla chaetica (Zhang et al., 2017). Thus, which receptor genes on the palps are required for locust vomiting? To answer this question, we first confirmed the cellular localization of several candidate ORs on the palps (Li et al., 2018a) through RNA *in situ* hybridization. As expected, OR12, OR17, OR19, OR21, and OR22 were expressed sparsely on the palps (Figures 2A–D, 5C). Based on the very few neurons labeled, it is likely that all 5 ORs were located in pb ORNs, with an expression pattern similar to that of OR2 (Zhang et al., 2017). No labeling was detected under the same conditions using sense probes of ORs as control (Figure 2A’–D’).

**FIGURE 3:**
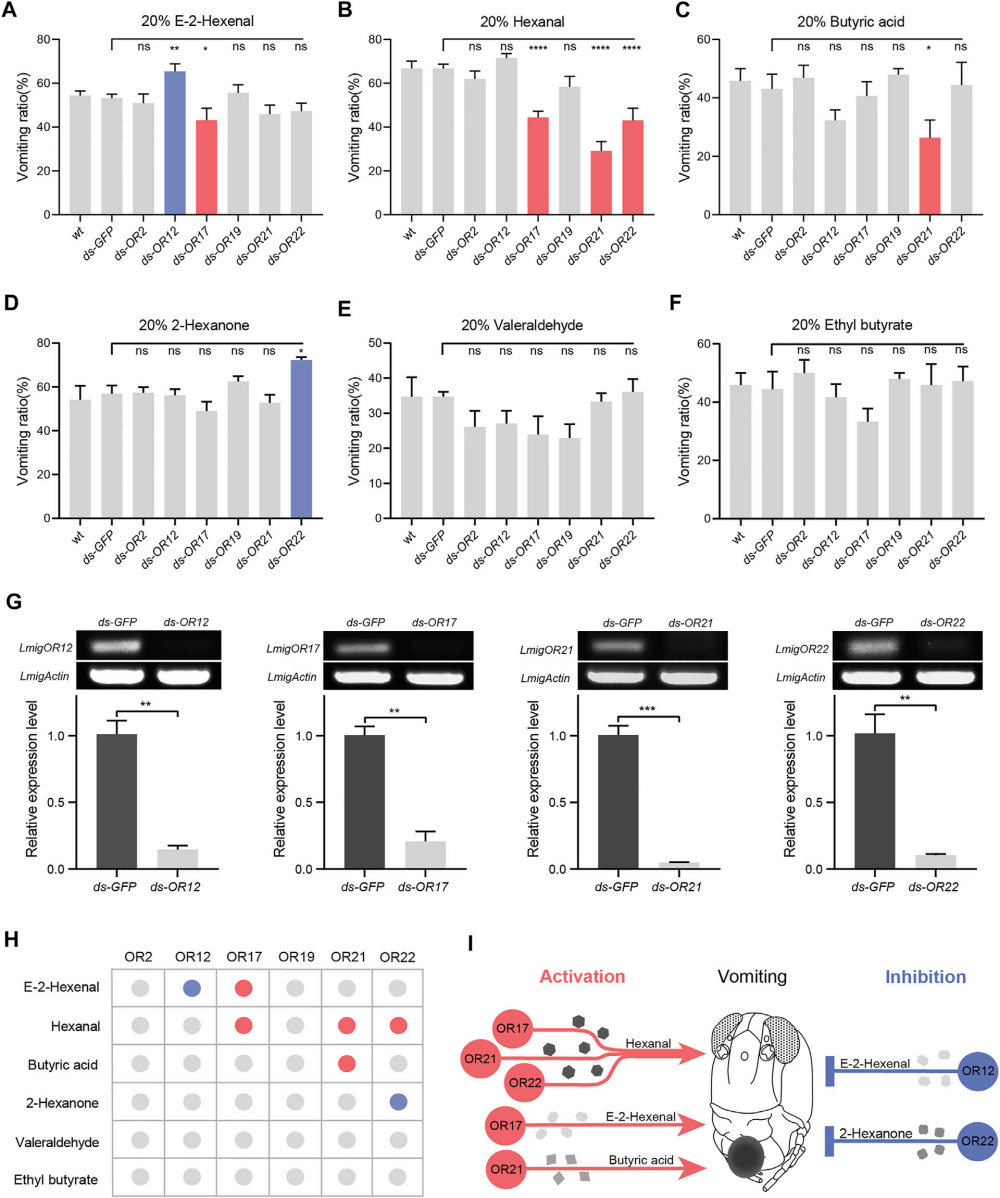
Odorant receptors are involved in the activation or inhibition of locust vomiting. **(A–F)** Vomiting response assays toward multiple compounds with wild-type locusts and locusts treated with dsRNA of GFP or an individual OR. *n* = 3–6 (24 locusts per replicate) for each assay. Statistical test: ordinary one-way ANOVA with uncorrected Fisher’s LSD test. **(G)** Effects of RNAi against OR12, OR17, OR21, and OR22, *n* = 3. Statistical test: unpaired two-tailed t-test. **(H)** Table listing the regulation modes of palpal ORs on locust vomiting response. The blue dot indicates inhibition, the orange dot indicates activation, and the gray dot indicates no response. **(I)** Schematic diagram of locust vomiting response regulated by ORs in two modes: inhibition and activation. For all analyses, statistical differences are represented as follows: ns, not significant, *p < 0.05, **p < 0.01, ***p < 0.001, and ****p < 0.0001. Data are represented as the mean ± SEM.

We next knocked down OR genes to identify candidate tuning receptors in locust vomiting. First, RNAi treatments of six OR genes on the palps led to inconsistent vomiting deficiency among six odorants (Figures 3A–F). Second, down-regulation of IRs expression also suppressed vomiting evoked by hexanal but not E-2-hexenal (Supplementary Figures S3A, B). In more detail, the vomiting ratio of dsOR12-injected locusts to E-2-hexenal was increased significantly, while that of dsOR17-injected locusts was reduced (Figure 3A). Knocking down one of the three ORs (OR17, OR21, and OR22) led to a lowered vomiting intensity to hexanal (Figure 3B). Similarly, knocking down OR21 resulted in significantly attenuated vomiting intensity to butyric acid (Figure 3C). Furthermore, knocking down OR22 led to enhanced vomiting intensity to 2-hexanone (Figure 3D). It is noteworthy that the expression of both ORs and IRs genes was substantially suppressed (Figure 3G and Supplementary Figures S2, S3C). In summary, a combinatorial pattern between odorants and ORs was established to demonstrate how the ligand–receptor complex influences vomiting reflex (Figures 3H,I): one odorant can be tuned by two distinct ORs that each triggers reverse behavioral output, and one OR is required for odor-specific behavioral performance. These results indicate that ORs participate in controlling locust vomiting through two modes: inhibition and activation.

### Parallel and Overlapping Patterns Between ORs in the Control of Vomiting

**FIGURE 4:**
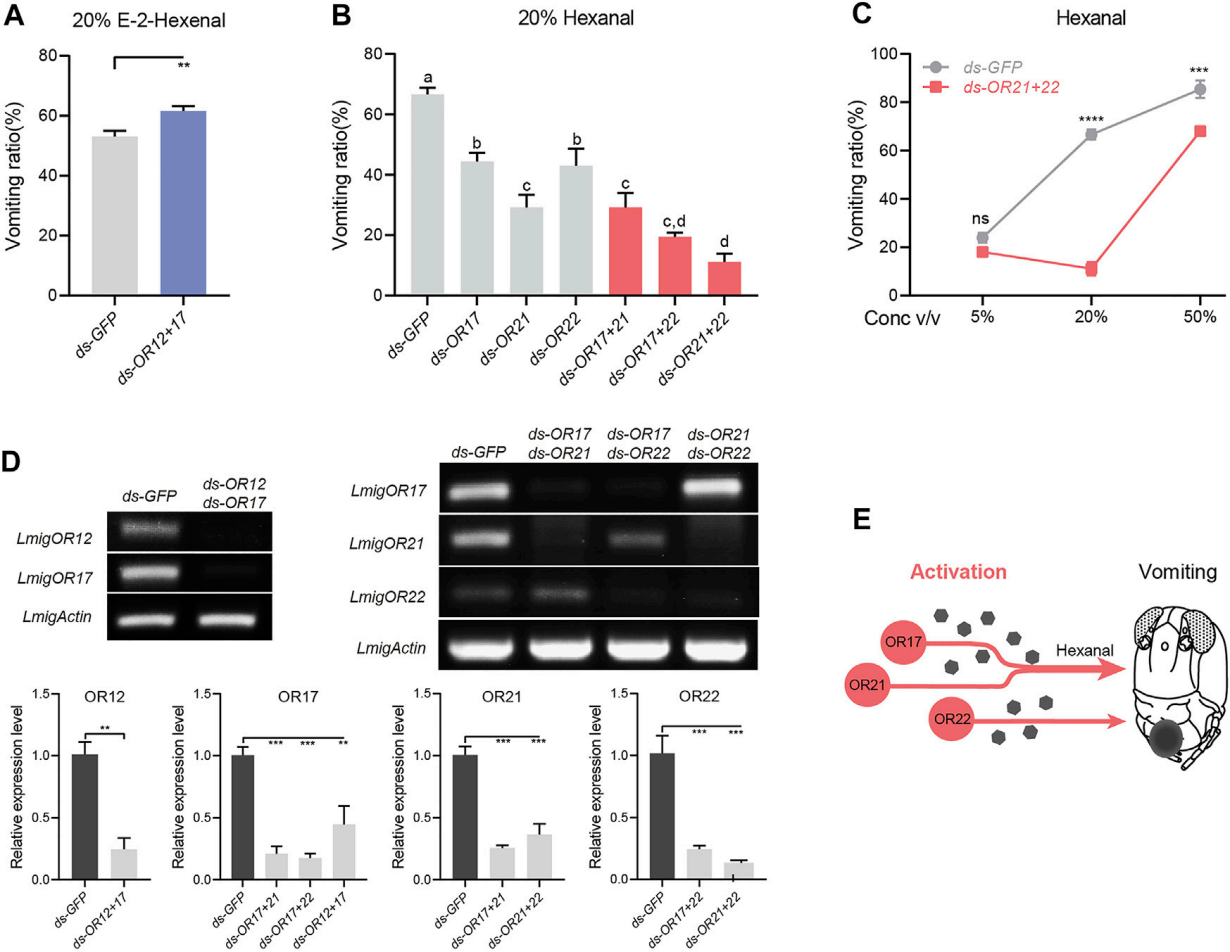
Parallel and overlapping patterns between ORs in the control of vomiting. **(A)** Vomiting response assays toward 20% v/v E-2-hexenal with locusts treated with ds RNA mix of OR12 and OR17. *n* = 5 (24 locusts per replicate) for each assay. Statistical test: unpaired two-tailed t-test. **(B)** Vomiting response assays toward 20% v/v hexanal with locusts treated with individual dsRNA or a mix of two ORs. *n* = 3–5 (24 locusts per replicate) for each assay. Statistical test: ordinary one-way ANOVA with uncorrected Fisher’s LSD test. **(C)** Vomiting response assays toward hexanal at concentrations of 5–50% v/v with locusts treated with dsRNA mix of OR21 and OR22. *n* = 3 (24 locusts per replicate) for each assay. Statistical test: two-way ANOVA with uncorrected Fisher’s LSD test. **(D)** Effects of RNAi against two ORs, *n* = 3. Statistical test: unpaired two-tailed t-test and ordinary one-way ANOVA with uncorrected Fisher’s LSD test. **(E)** Schematic model showing that OR17, OR21, and OR22 function in both overlapping and parallel manners. For all analyses, statistical differences are represented as follows: ns, not significant, **p < 0.01, ***p < 0.001, and ****p < 0.0001. Different letters indicate statistically significant differences between groups. Data are represented as the mean ± SEM.

OR12 and OR17 were revealed to have distinct roles in modulating vomiting in response to 20% E-2-hexenal. The question is which signaling weighs more when both are activated. Dual injections of dsRNA against OR12 and OR17 led to mildly enhanced vomiting intensity to E-2-hexenal (Figure 4A), although the enhancement was lower than that with OR12 RNAi alone (Figure 3A). Therefore, OR12 plays a dominant role in suppressing locust vomiting induced by E-2-hexenal. Similarly, since all three ORs, OR17, OR21, and OR22, were indispensable for the triggering of vomiting in response to hexanal, we proposed the logical assumption that these ORs function together. The expected scenarios include three actions: three ORs act on an overlapped pathway, or on three parallel pathways, or any two ORs act on an overlapped pathway, and the third OR acts on a parallel pathway. Dual RNAi against any two of the three ORs led to a significantly lower response to hexanal, while dual RNAi against OR21 and OR22 led to the smallest vomiting ratio among all pairings (Figure 4B). Notably, the co-suppression of OR17 and OR21 led to no additional negative effects compared with a single RNAi against OR21 alone (Figure 4B), which suggests that the two ORs may function in an overlapping pathway. Furthermore, the dose–response curve when both OR21 and OR22 were silenced revealed significantly attenuated vomiting intensity at higher doses than when locusts were injected with dsGFP (Figure 4C). Notably, all dual RNAi assays showed strong silencing efficiency (Figure 4D). Together, these findings revealed that the hexanal-dependent vomiting response relied on three ORs that function in both overlapping and parallel manners (Figure 4E).

### OR12 Tunes the Response to E-2-Hexenal in Olfactory Neurons in a Basiconic Sensillum

**FIGURE 5:**
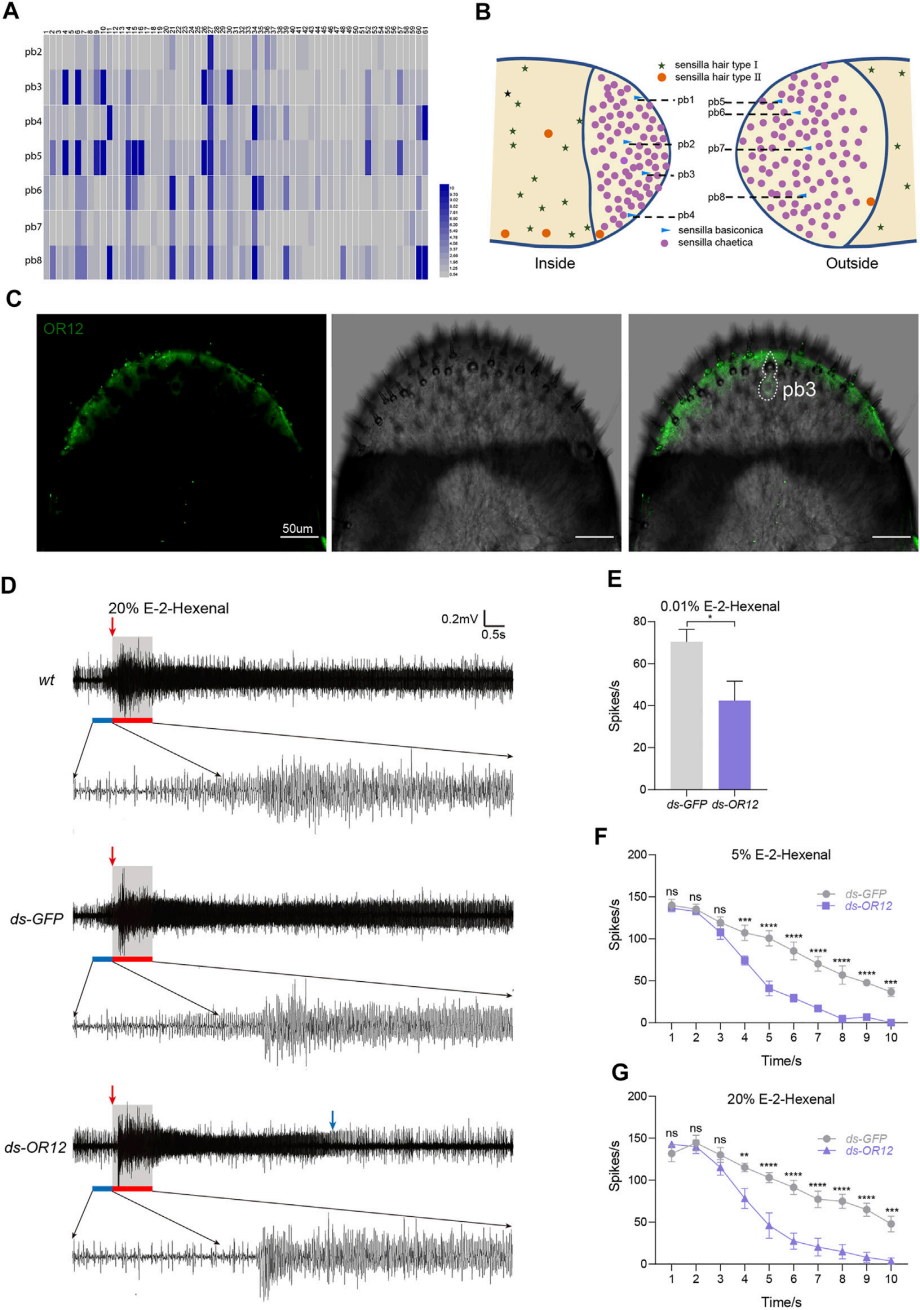
OR12 tunes the response to E-2-hexenal in olfactory neurons in a basiconic sensillum. **(A)** Coding of odors by the basiconic sensilla on the maxillary palp. The top numbers (1–61) represent paraffin oil and 60 characteristic odors used for stimulation. On the left, pb2-pb8 are 7 subtypes of basiconic sensilla on the maxillary palp. The color code from blue to red indicates the recovery time from 0 to 10 s. The recovery time is the duration of continuous excitation of neurons in the basiconic sensilla after stimulation. *n* = 6–7 palps per odor. **(B)** Schematic diagram of the distributions of the basiconic sensilla on the maxillary palp. **(C)** Whole-mount fluorescence *in situ* hybridization (WM-FISH) using OR12-specific DIG-labeled antisense RNA probes. Cells bearing OR12 were visualized by green fluorescence. The white dashed line marks neurons expressing OR12 and the corresponding palp basiconic sensillum (pb3). Scale bars: 50 µm. **(D)** Representative traces showing the response of pb3 to 20% v/v E-2-hexenal in wild-type locusts and locusts treated with dsRNA of GFP or OR12. The blue bar marks 0.5 s of spontaneous activity before stimulation. The red bar marks stimulus delivery and duration (1 s). The red arrow indicates the point at which the stimulus begins. The blue arrow indicates the point at which the reaction ends. **(E)** Quantification of mean changes of all spikes in 1 s before and after 0.01% (v/v) E-2-hexenal stimulus. *n* = 6–10 sensilla. Statistical test: unpaired two-tailed t-test. **(F,G)** Quantification of mean changes of spikes per 1 s within 10 s after 5% **(F)** and 20% **(G)** v/v E-2-hexenal stimulus, △spikes = the number of spikes per 1 s within 10 s after stimulation-the number of spontaneous spikes in 1 s before stimulation. *n* = 6–10 sensilla. Statistical test: two-way ANOVA with uncorrected Fisher’s LSD test. For all analyses, statistical differences are represented as follows: ns, not significant, *p < 0.05, **p < 0.01, ***p < 0.001, and ****p < 0.0001. Data are represented as the mean ± SEM.

The dome region of the maxillary palp is covered with sensilla chaetica (roughly 98%) and sensilla basiconica (2%, approximately 8) (Blaney et al., 1971; Blaney, 1977; Jin et al., 2005). To functionally distinguish each palp sensilla basiconica (pb), extensive extracellular recordings in a single sensillum were performed *via* challenging a panel of odorants (Supplementary Table S5). We quantified how long the activated spiking decays into the basal level after the odorant stimulation was triggered (termed as the decaying duration). This strategy was chosen because of the large number of ORNs (∼15) inside the pbs (Jin et al., 2005), which dampens the clear separation of each spike. We uncovered eight functional basiconica subtypes (pb1–pb8) on the dome based on physiological response patterns (Figure 5A). Topologically, pb1, pb2, pb3, and pb4 were distributed from top to bottom on the outer surface, while pb5, pb6, pb7, and pb8 were located from top to bottom on the inner surface (Figure 5B). Thus, each pb can be accessed *via* diagnostic chemicals. The next step was to link the OR repertoire with pb subtypes. In this study, we located one OR12-positive ORN in the pb3 subtype by using whole-mount FISH (Figure 5C).

We next asked whether OR12 tunes the electrophysiological response to E-2-hexenal in pb3 sensilla. Control pb3 neurons were activated with long-lasting firing in response to 20% E-2-hexenal, while the excitation duration in dsOR12-injected locusts was much shorter (Figure 5D). We observed no distinguishable difference of decaying dynamics within 10 s after stimulation between dsGFP-injected locusts and wild-type locusts (Supplementary Figure S4E). Knocking down OR12 maintained a similar decaying response in the first 3 s after stimulation, while it decayed significantly faster (sharper slope) since the 4^th^ second (Figure 5G). These results suggest that OR12 can prolong the firing state of pb3 neurons to E-2-hexenal.

In addition, we also tested the electrophysiological responses of pb3 neurons to low concentrations of E-2-hexenal. A lower dose of E-2-hexenal (0.01%) strongly activated pb3 neurons in both control and dsGFP-injected locusts (Supplementary Figure S4A), and this physiological activation was OR12 dependent (Figure 5E). Wild-type pb3 ORNs were intensely activated with a prolonged response up to 10 s after the initial stimulation of 5% E-2-hexenal (Supplementary Figure S4B). In contrast, the suppression of OR12 led to a significantly faster decaying response (Figure 5F), which was similar to that at 20% dilutions. Together, these results show that OR12 was required for slowed decaying activity in pb3 sensilla in response to E-2-hexenal.

### Two-Order Olfactory Projection Patterns Underlying Vomiting Signaling in the Brain

**FIGURE 6:**
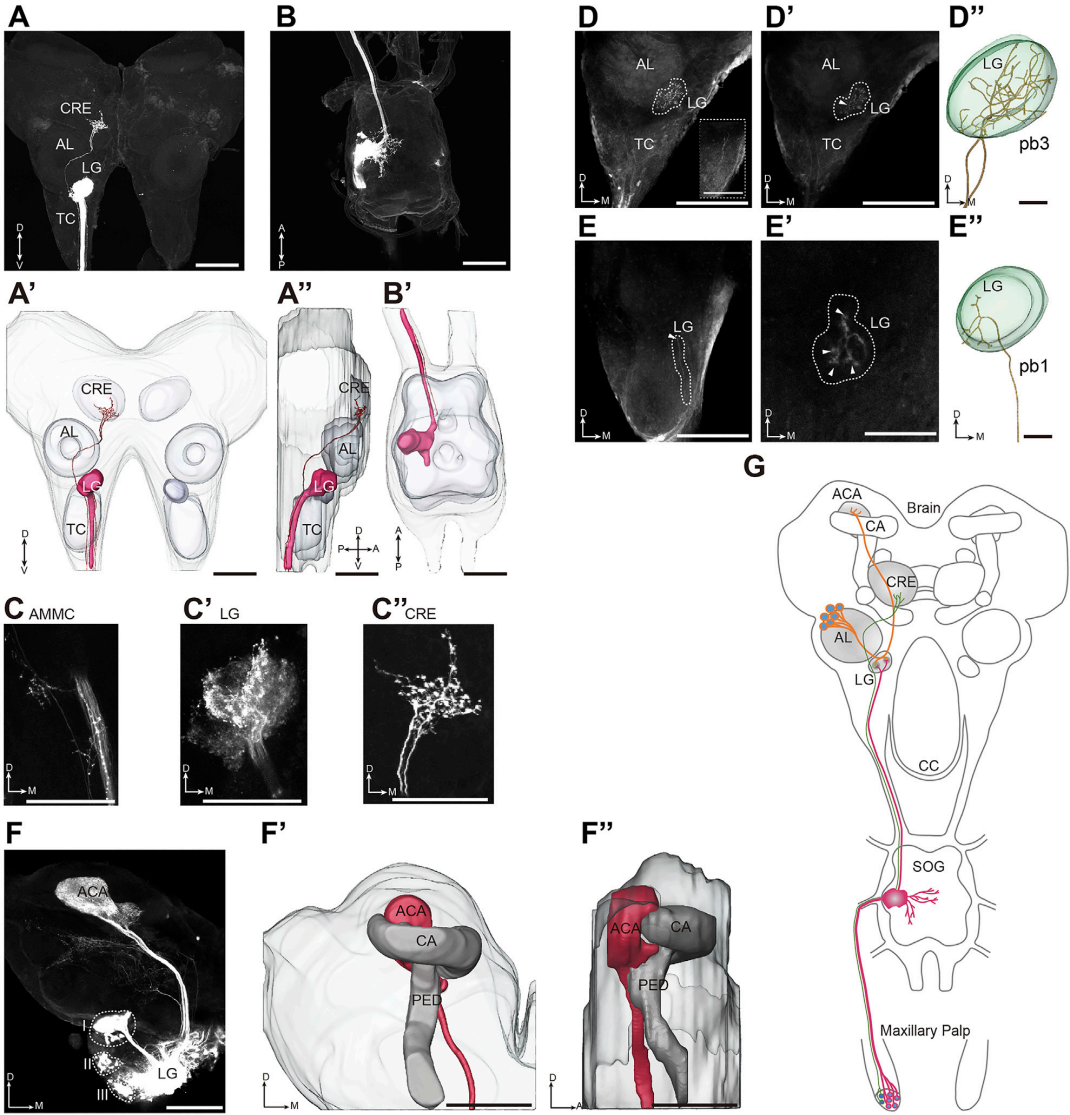
Two-order olfactory projection patterns underlying vomiting signaling in the brain. **(A,B)** Confocal images of the brain **(A)** and the SOG **(B)** with stained afferents originating from the maxillary palp. **(A′,A′′)** Three-dimensional reconstruction of the stained neurons shown in **(A)** (frontal and lateral views, respectively). **(B′)** Three-dimensional reconstruction of the stained neurons shown in **(B)**. **(C-C′′)** Terminals of sensory neuron axons originating from the maxillary palp innervate the AMMC **(C)**, the LG **(C′),** and the CRE **(C′′)**. **(D,D′)** Confocal images of the brain with stained afferents originating from pb3. The dotted box in **(D)** marks two plexi of axons originating from the pb3 neurons running through the ipsilateral TC. The dotted line marks the projected region in the brain, and the white arrow shows the axonal entry point into the LG. **(D′′)** Three-dimensional reconstructed images of the ORN axons shown in **(D)**. **(E,E′)** Confocal images of the brain with stained afferents originating from pb1. The projected region is marked by the dotted line. The white arrow in **(E)** shows the axonal entry point into the LG, and the white arrows in **(E′)** show four branches of the axons in the LG. **(E′′)** Three-dimensional reconstructed images of the ORN axons shown in **(E)**. **(F)** Confocal images of the main glomerular lobe-calycal pathway obtained by selective staining of PNs linked to the LG. The LG is specifically connected to the ACA of the MB. **(F′,F′′)** Three-dimensional reconstruction showing the overall projection pathway of the PNs in the LG (frontal and lateral views, respectively). The dotted line marks the soma clusters of the PNs. **(G)** Schematic representation of the neural circuit of locust vomiting. SOG, suboesophageal ganglion; CC, circumoesophageal connective; TC, tritocerebrum; DC, deutocerebrum; PC, protocerebrum; AL, antennal lobe; LG, lobus glomerulatus; CRE, crepine; AMMC, antennal mechanosensory and motor center; MB, mushroom body; CA, calyx; ACA, accessory calyx; PED, pedunculus; A, anterior; D, dorsal; M, medial; P, posterior; and V, ventral. Scale bars: 200 µm in **(A-A′′)**, **(B-B′′)**, **(D-D′)**, **(E-E′),** and **(F-F′′)**; 100 µm in **(C-C′′)** and the dotted box of **(D)**; and 50 µm in **(D′′)** and **(E′′)**.

OR17, OR21, and OR22 are expressed in both the antennae and palps (Wang et al., 2015; Li et al., 2018a); however, palps, but not antennae were required in vomiting (Figures 1E–G and Supplementary Figure S1A). We proposed that this differential tissue requirement is based on the fact that ORNs in the antennae and palps have different projection patterns in the first relaying center in the brain. Next, we sought to explore the olfactory neural pathways that mediated locust vomiting. We first characterized the morphology of the central nervous system by neuropil immunostaining (Supplementary Figures S5B,C) and further reconstructed the three-dimensional structure of both the brain and the suboesophageal ganglion (SOG) using Amira software (Supplementary Figures S5B’,C’). Different brain areas were registered based on their counterparts in the desert locust (von Hadeln et al., 2018). Next, we focused on the first olfactory relaying center in the brain, the antennal lobe (AL), and the additional lobus glomerulatus (LG). To trace the olfactory circuitry that originates from pb ORNs and terminates in the olfactory processing center, *in vivo* anterograde staining of the maxillary and labial neuronal afferents revealed a distinct tract that ascended from the SOG through the circumoesophageal connective (CC) into the brain (Figure 6 and Supplementary Figure S6). Axonal bundles of the maxillary palp sensory neurons entered the SOG *via* the ipsilateral maxillary nerve and further innervated the LG and slightly innervated the antennal mechanosensory and motor center (AMMC) (Figures 6A,A’,A’’,B,B’,C,C’). In addition, an independent neural branch passed over the LG-AL complex and projected into the crepine (CRE) area with abundant local arborizations (Figures 6A,A’,A’’,C’’). However, this independent branching circuit in the maxillary palp was lacking in the projection areas from axonal bundles of the labial palp sensory neurons, although the SOG and LG areas were predominantly innervated (Supplementary Figure S6).

We also investigated the central projections of ORNs under individual basiconic sensillum of the maxillary palp. Regarding pb3, two axonal bundles ran through the ipsilateral tritocerebrum (TC) without bifurcation and entered into the medial part of the LG with a converged bundle. After entering the LG, the primary tract of axons dispersed to dozens of local arborizations (Figures 6D,D’,D’’). Due to the large number of ORNs and the dense axon projection patterns, it was impossible to distinguish between the central projections of individual neurons originating from pb3. For pb1, a bundle of axons ascended through the ipsilateral TC and then entered the LG, where the axons dispersed into four branches and each of them arborized in distinct areas (Figures 6E,E’,E’’).

Given that ORNs of the sensilla basiconica project into the LG, tracing dye was injected into the mass LG region to allow the direct trace of secondary PNs. At least three PN populations that relay information from the LG into the higher accessory calyx (ACA) of the posterior mushroom body (MB) were marked (Figure 6F,F’,F’’). Moreover, a small bundle of axons branched off from the primary tract and innervated the anterolateral region of the brain (Figure 6F). Taken together, these anatomical data suggest that ORNs originating from the sensilla basiconica in the maxillary palp principally projected into the LG *via* the CC, and the PNs in the LG further projected into the ACA of the MB to probably mediate the vomiting response (Figure 6G).

## Discussion

In this study, we established an olfaction-based vomiting model using migratory locusts. Compared with mammals, locusts are small in size, fast in reproduction, and simple in physiological and neural structure. More importantly, locusts vomit sensitively to chemicals, which is convenient for phenotype analysis. In addition, multiple experimental techniques such as RNAi, CRISPR-based genome editing, *in situ* hybridization, and electrophysiology have been well applied in locusts, largely facilitating the exploration of the mechanism of vomiting at molecular and cellular levels.

### The Combinatorial Coding Strategy of Olfaction in Vomiting

Here, we identified a panel of active odorants that evoke intense vomiting reflex, as well as the cognate ORs *via* RNAi. Most dominantly, E-2-hexenal and hexanal were encoded by different combinations of palp ORs and IRs to trigger the vomiting reflex. Intriguingly, antennae were not required for vomiting, since stimulation toward antennae failed to evoke any vomiting in palp-less locusts. A previous report showed that most palp ORs involved in this study were also detected on antennae (Li et al., 2018a), thus presenting one question of how ORNs expressing identical ORs on different olfactory organs contribute to vomiting distinctly. One possibility is that ORNs have different projection patterns in the primary olfactory center. In locusts, the ORNs from the antennae and palps (the maxillary palp and the labial palp) send their axons to the AL and LG, respectively (Ignell et al., 2000). Similar projection patterns were also reported in other insects, such as beetles and mosquitoes. For example, Dippel et al. (2016) reported that in *Tribolium castaneum*, LG receives olfactory inputs from the palps while AL is innervated with axonal projections from antennal ORNs. In mosquitos, although the ORNs from the antenna and the maxillary palp all project to the AL, their projection areas in the AL are separate. There are notably three glomeruli in the AL that are innervated by the maxillary palp (Kwon et al., 2006; Riabinina et al., 2016; Younger et al., 2020). It is worth noting that mosquito CO_2_-sensitive ORNs on the maxillary palp but not on the antennae are required to localize humans (Younger et al., 2020). Together, separated projection patterns between olfactory organs in the brain might explain their different roles in behavioral outputs. This study supported this argument and expanded the evidence that the PNs from the LG target higher brain regions of the accessory calyx (ACA). However, whether the AL sends projections into the ACA should be checked. Thus, the two olfactory circuitries modulate different physiological activities, and the palp ORN-LG-ACA axis may be vomiting specific. Nevertheless, functional connections between palp ORNs and LG/ACA areas deserve more physiological evaluation; for example, the activity of LG areas should be determined when the palps are stimulated with various odorants.

### Distinct ORs Modulate the Reverse Effects on Vomiting From One Chemical

Canonically, individual OR mediates specific behavior that is evoked with certain odorants (Hallem and Carlson, 2006; Stensmyr et al., 2012; Ronderos et al., 2014); however, this study presented a novel functional model of OR coding logic: one odorant can be tuned by two distinct ORs that each triggers reverse behavioral output. On the one hand, E-2-hexenal evoked intense vomiting, which was combinatorially coded by stimulative OR17 and suppressive OR12. On the other hand, one OR is required for odor-specific behavioral tendencies: the suppression of OR22 attenuated the vomiting caused by hexanal while promoting this response to 2-hexanone. This coding pattern greatly enhanced the diversity and dimension of the olfactory system in sensing external cues. However, how the activation of different ORN subtypes leads to reversed behavioral outputs remains unclear. To visualize the axonal arborizations of palp ORNs on the SOG and LG, as well as further projections in the ACA, each ORN subtype that expresses different tuning ORs must be genetically marked with a membrane-bound fluorescent marker to trace the complete projection into the SOG and LG. This step will greatly improve the precision of comparing the detailed innervation differences among ORN subtypes; different innervation patterns in the SOG and LG may produce new synaptic connections that result in the final promotion or suppression of vomiting. Another explanation may come from differences in firing patterns between ORN subtypes. Although ORNs are stimulated with odorants of identical dilutions, their firing characteristics may vary; thus, the brain neurons are modulated differentially. In addition, nonsynaptic direct communications between grouped neurons in an olfactory sensillum are observed to modulate behavior (Su et al., 2012). Currently, no colocalization evidence among ORs in the same ORN or same sensillum is presented.

### Coding Logic Among Odorant Receptors

Specifically, OR17/OR21/OR22 were associated to tune the response to hexanal, and the suppression of each OR reduced the vomiting ratio. Knocking down certain OR combinations intensely alleviated the vomiting reflex. One possible trafficking logic is that OR17 and OR21 mediate the response to hexanal through one signaling pathway. Another scenario suggests that OR17 and OR21 are coexpressed on the same neuron or that individual ORNs housing these two ORs are in the same signaling pathway contributing to hexanal-evoked vomiting. The first case might be confirmed by coexpression *via* two-color *in situ* RNA hybridization (Xu et al., 2013), or simultaneous expression of OR17 and OR21 in a heterologous system may gain more support to challenge the traditional view that two specific ORs are mostly not expressed on the same ORN (Vosshall et al., 2000; Larsson et al., 2004; Benton et al., 2006). Also, we wondered how OR12 outperformed OR17 to gain the ultimate control of vomiting. One possibility is that the interaction between ORNs expressing distinct ORs attenuates the ultimate firing output, and this process may occur within one sensilla or LG. This argument requires the visualization of ORNs using transgenic gene labeling to reveal cellular interaction in pbs and also the projection patterns from individual ORNs in LG.

Another olfactory receptor family, IRs, is also involved in hexanal-induced vomiting. A previous study revealed that both ORs and IR8a are necessary for palp opening response (POR) using hexanal (Zhang et al., 2017). Thus, this raises another question: how IRs and ORs are accompanied to control vomiting and where this process occurs. First, it is laudable to dissect the central projection pattern of IR-expressing ORNs, for example, whether these two olfactory pathways potentially interact at LG. In addition, no projections in the ALs were found from palp ORNs expressing IR8a and IR25a, thus providing more evidence that the ALs are not required to receive inputs from palp IRs + ORNs to trigger vomiting. Obviously, IR8a and IR25a were abundantly expressed on the antennae, indicating again that the AL and LG are functionally independent in vomiting.

## Data Availability

The original contributions presented in the study are included in the article/Supplementary Material, further inquiries can be directed to the corresponding author.
