# Supplementary material for: Odor-Induced Vomiting Is Combinatorially Triggered by Palp Olfactory Receptor Neurons That Project to the Lobus Glomerulatus in Locust Brain
Source: Front Physiol. 2022 Apr 20;13:855522. doi: 10.3389/fphys.2022.855522 (PMC9065551; doi:10.3389/fphys.2022.855522)
Supplement: Supplementary file 1 [file DataSheet1.docx]

Table S1 The primers for probe preparation.

| Gene ID | Forward Primer（5’ to 3’） | Reverse Primer（5’ to 3’） |
| --- | --- | --- |
| *LmigOR12* | CTCAGTTGCGGATGCAGCCA | AGCTCGGATCTGTAGTGGGT |
| *LmigOR17* | TCTACCTCTTCAGCACCGAC | CCACACCAGCAGTAGAGGAA |
| *LmigOR19* | CTTACGTCTATAACAGCTGGAA | ACTCATCTGACGTAATATAGCG |
| *LmigOR21* | ACCTCTCGCTGTACGTGTC | GTTCGTGCCCGCCATAGCAAT |
| *LmigOR22* | TGGTACGCCTCCCCGCTCA | TCTCCAGTTCGACGCCCAGGT |

Table S2 The primers for dsRNA preparation.

| Gene ID/ GenBank accession number | Forward Primer（5’ to 3’） | Reverse Primer（5’ to 3’） |
| --- | --- | --- |
| *LmigOR2*/ KP843285 | GGATCCTAATACGACTCACTATAGGCACCTACCACCTGCTGTACG | GGATCCTAATACGACTCACTATAGGCTACTGTGATCTCCACCGGC |
| *LmigOR12*/KP843312 | GGATCCTAATACGACTCACTATAGGCAGAGCGACTTCAAACC | GGATCCTAATACGACTCACTATAGGGCAAGGCTACACTCAAATAC |
| *LmigOR17*/KP843365 | GGATCCTAATACGACTCACTATAGGTCTACCTCTTCAGCACCGAC | GGATCCTAATACGACTCACTATAGGCCACACCAGCAGTAGAGGAA |
| *LmigOR19*/KP843237 | GGATCCTAATACGACTCACTATAGGATTATGGACCACCAGGAA | GGATCCTAATACGACTCACTATAGGGTAGGAGTAAGAAGCGTTG |
| *LmigOR21*/KP843321 | GGATCCTAATACGACTCACTATAGGTGGTTCCCGTTCGACCCAGT | GGATCCTAATACGACTCACTATAGGGCCAGTGTCCCAAAGTCGCTCT |
| *LmigOR22*/KP843252 | GGATCCTAATACGACTCACTATAGGGACGGCGGTGAAATGGTG | GGATCCTAATACGACTCACTATAGGGGCGGAGTAAAGCGTAGAATG |
| *LmigIR8a*/ KP843224 | GGATCCTAATACGACTCACTATAGGAGTCTGGCATCTCTATCGTGAT | GGATCCTAATACGACTCACTATAGGAACATCTCTCCAACCTCAGTCA |
| *LmigIR25a*/KP843219 | GGATCCTAATACGACTCACTATAGGAAGTCAAACCCTCAGTGCCAG | GGATCCTAATACGACTCACTATAGGTCCTCCCTGTGGCGTTAAGGATG |
| *GFP* | GGATCCTAATACGACTCACTATAGGCACAAGTTCAGCGTGTCCG | GGATCCTAATACGACTCACTATAGGGTTCACCTTGATGCCGTTC |

Table S3 The primers for RT-qPCR and semi-quantitative RT-PCR.

| Gene ID | Forward Primer（5’ to 3’） | Reverse Primer（5’ to 3’） |
| --- | --- | --- |
| *LmigActin* | AAATCTGGCACCACACCTTC | GGTTCAATGGGGCTTCAGTC |
| *LmigOR2* | CCACCTACCACCTGCTGTA | GGCTGCGATATTCTCGTTCAA |
| *LmigOR12* | AAATCTGGCACCACACCTTC | GGTTCAATGGGGCTTCAGTC |
| *LmigOR17* | CGAGAATACAATCCGAAT | ATAAACTGCCTCTGATATG |
| *LmigOR19* | GTAGTCCTTGTTCAGTTC | CAGTATATGAATAGTTGAAGAATA |
| *LmigOR21* | CGGTCTCCATTTGCATCACC | ATCAATTAGTTCGTGCCCGC |
| *LmigOR22* | AGGAAGTGATGGGAATCT | GCTCTTCGTCGTTGTAAT |
| *LmigIR8a* | CCAGAGCCGCATCAACTAC | CCCATACCCTGTACCTAGACTG |
| *LmigIR25a* | AAATGACAGCCTGAGTGAAGTCG | ATCCAGCTTCAGTCATGGCTTG |
| *LmigOrco*  /KP843368 | CGCTCCGCCATCAAGTAC | CGTCCACCGAGTCAATCTTG |

Table S4 The chemical compounds for vomiting behavior experiment.

| Odorant | Molecular Formula | CAS | Manufacturer |
| --- | --- | --- | --- |
| Paraffin oil |  | 8042-47-5 | SIGMA-ALDRICH |
| **Alcohol** |  |  |  |
| 1-Pentanol | C_5_H_12_O | 71-41-0 | ALDRICH |
| 3-Methyl-1-butanol | C_5_H_12_O | 123-51-3 | SIGMA-ALDRICH |
| cis-3-Hexen-1-ol | C_6_H_12_O | 928-96-1 | ALDRICH |
| 1-Heptanol | C_7_H_16_O | 111-70-6 | ALDRICH |
| DL-sec-Phenethyl alcohol | C_8_H_10_O | 98-85-1 | ACROS ORGANICS |
| 1-Octen-3-ol | C_8_H_16_O | 3391-86-4 | ALDRICH |
| cis-3-Nonen-1-ol | C_9_H_18_O | 10340-23-5 | ALDRICH |
| 1-Nonanol | C_9_H_20_O | 143-08-8 | TCI |
| 1-Hexanol | C_6_H_14_O | 111-27-3 | SIGMA-ALDRICH |
| 2-Butyl-1-octanol | C_12_H_26_O | 3913-02-8 | ALDRICH |
| Phytol | C_20_H_40_O | 150-86-7 | ALDRICH |
| **Ester** |  |  |  |
| Methyl isovalerate | C_6_H_12_O_2_ | 556-24-1 | ALDRICH |
| Ethyl butyrate | C_6_H_12_O_2_ | 105-54-4 | ALDRICH |
| Butyl acrylate | C_7_H_12_O_2_ | 141-32-2 | ALDRICH |
| Ethyl heptanoate | C_9_H_18_O_2_ | 106-30-9 | ALDRICH |
| Ethyl caprylate | C_10_H_20_O_2_ | 106-32-1 | ALDRICH |
| Nonyl acetate | C_11_H_22_O_2_ | 143-13-5 | SAFC |
| Ethyl caprate | C_12_H_24_O_2_ | 110-38-3 | ALDRICH |
| Ethyl dodecanoate | C_14_H_28_O_2_ | 106-33-2 | Alfa Aesar |
| Lauryl methacrylate | C_16_H_30_O_2_ | 142-90-5 | ALDRICH |
| Ethyl myristate | C_16_H_32_O_2_ | 124-06-1 | ALDRICH |
| Dibutyl phthalate | C_16_H_22_O_4_ | 84-74-2 | Fluka |
| **Ketone** |  |  |  |
| 2-Hexanone | C_6_H_12_O | 591-78-6 | SIGMA-ALDRICH |
| 2-Heptanone | C_7_H_14_O | 110-43-0 | ACROS ORGANICS |
| 6-Methyl-5-hepten-2-one | C_8_H_14_O | 110-93-0 | ALDRICH |
| Acetophenone | C_8_H_8_O | 98-86-2 | SIGMA-ALDRICH |
| 2-Octanone | C_8_H_16_O | 111-13-7 | ALDRICH |
| 2,6,6-Trimethyl-2-cyclohexene-1,4-dione | C_9_H_12_O_2_ | 1125-21-9 | ACROS ORGANICS |
| (+)-Carvone | C_10_H_14_O | 2244-16-8 | Fluka |
| **Aldehyde** |  |  |  |
| E-2-Pentenal | C_5_H_8_O | 1576-87-0 | ALDRICH |
| Valeraldehyde | C_5_H_10_O | 110-62-3 | ALDRICH |
| E,E-2,4-Hexadienal | C_6_H_8_O | 142-83-6 | ALDRICH |
| 2-Methyl-2-pentenal | C_6_H_10_O | 623-36-9 | ALDRICH |
| E-2-Hexenal | C_6_H_10_O | 6728-26-3 | ALDRICH |
| Hexanal | C_6_H_12_O | 66-25-1 | ALDRICH |
| Benzaldehyde | C_7_H_6_O | 100-52-7 | SIGMA-ALDRICH |
| Heotaldehyde | C_7_H_14_O | 111-71-7 | ALDRICH |
| E-2-Octenal | C_8_H_14_O | 2548-87-0 | ALDRICH |
| Octanal | C_8_H_16_O | 124-13-0 | ALDRICH |
| Nonanal | C_9_H_18_O | 124-19-6 | TCI |
| Decanal | C_10_H_20_O | 112-31-2 | SIGMA |
| **Acid** |  |  |  |
| Butyric acid | C_4_H_8_O_2_ | 107-92-6 | ALDRICH |
| Octanoic acid | C_8_H_16_O_2_ | 124-07-2 | ALDRICH |
| **Others** |  |  |  |
| 2,5-Dimethylpyrazine | C_6_H_8_N_2_ | 123-32-0 | TCI |
| Phenylacetonitrile | C_8_H_7_N | 140-29-4 | Fluka |
| Decane | C_10_H_22_ | 124-18-5 | SIGMA-ALDRICH |

Table S5 The chemical compounds for single sensillum recording.

| Number | Odorant | Molecular Formula | CAS | Manufacturer |
| --- | --- | --- | --- | --- |
| 1 | Paraffin oil |  | 8042-47-5 | SIGMA-ALDRICH |
| 2 | E-2-hexen-1-ol | C_6_H_12_O | 928-95-0 | ALDRICH |
| 3 | Linalool | C_10_H_18_O | 78-70-6 | ALDRICH |
| 4 | E-2-Hexenal | C_6_H_10_O | 6728-26-3 | ALDRICH |
| 5 | 2,6,6-Trimethyl-2-cyclohexene-1,4-dione | C_9_H_12_O_2_ | 1125-21-9 | ACROS ORGANICS |
| 6 | E,E-2,4-Hexadienal | C_6_H_8_O | 142-83-6 | ALDRICH |
| 7 | E,Z-2,6-nonadienal | C_9_H_14_O | 557-48-2 | ALDRICH |
| 8 | Decane | C_10_H_22_ | 124-18-5 | SIGMA-ALDRICH |
| 9 | cis-3-Nonen-1-ol | C_9_H_18_O | 10340-23-5 | SAFC |
| 10 | 2,4-Heptadienal | C_7_H_10_O | 224-328-0 | SIGMA-ALDRICH |
| 11 | 1-Octen-3-ol | C_8_H_16_O | 3391-86-4 | ALDRICH |
| 12 | cis-3-Hexen-1-ol | C_6_H_12_O | 928-96-1 | ALDRICH |
| 13 | 1-Hexanol | C_6_H_14_O | 111-27-3 | SIGMA-ALDRICH |
| 14 | Hexanal(Caproaldehyde) | C_6_H_12_O | 66-25-1 | ALDRICH |
| 15 | Valeraldehyde | C_5_H_10_O | 110-62-3 | ALDRICH |
| 16 | Butyraldehyde | C_4_H_8_O | 123-72-8 | ALDRICH |
| 17 | Dodecane | C_12_H_26_ | 112-40-3 | ALDRICH |
| 18 | 2-Heptanone | C_7_H_14_O | 110-43-0 | ACROS ORGANICS |
| 19 | 2-Methyl-2-pentenal | C_6_H_10_O | 623-36-9 | ALDRICH |
| 20 | Hydroxyacetone | C_3_H_6_O_2_ | 116-09-6 | ALDRICH |
| 21 | Octanal | C_8_H_16_O | 124-13-0 | ALDRICH |
| 22 | Butyl acetate | C_6_H_12_O_2_ | 123-86-4 | SIGMA-ALDRICH |
| 23 | Methyl isovalerate | C_6_H_12_O_2_ | 556-24-1 | ALDRICH |
| 24 | E-2-Octenal | C_8_H_14_O | 2548-87-0 | ALDRICH |
| 25 | 2-Hexanone | C_6_H_12_O | 591-78-6 | SIGMA-ALDRICH |
| 26 | E-2-Pentenal | C_5_H_8_O | 1576-87-0 | ALDRICH |
| 27 | Decanal | C_10_H_20_O | 112-31-2 | SIGMA |
| 28 | 2-Ethylhexanoic acid | C_8_H_16_O_2_ | 149-57-5 | TCI |
| 29 | Butyl Propionate | C_7_H_14_O_2_ | 590-01-2 | TCI |
| 30 | 1-Nonanol | C_9_H_20_O | 143-08-8 | TCI |
| 31 | 2,5-Dimethylpyrazine | C_6_H_8_N_2_ | 123-32-0 | TCI |
| 32 | 2-Ethyl-1-hexanol | C_8_H_18_O | 104-76-7 | ALDRICH |
| 33 | 1-Pentanol | C_5_H_12_O | 71-41-0 | ALDRICH |
| 34 | Nonanal | C_9_H_18_O | 124-19-6 | TCI |
| 35 | Nonyl acetate | C_11_H_22_O_2_ | 143-13-5 | SAFC |
| 36 | cis-3-Hexenyl acetate | C_8_H_14_O_2_ | 3681-71-8 | TCI |
| 37 | Phytol | C_20_H_40_O | 150-86-7 | ALDRICH |
| 38 | Ethyl undecanoate | C_13_H_26_O_2_ | 627-90-7 | ALDRICH |
| 39 | Ethyl nonanoate | C_11_H_22_O_2_ | 123-29-5 | ALDRICH |
| 40 | Lauryl methacrylate | C_16_H_30_O_2_ | 142-90-5 | ALDRICH |
| 41 | Ethyl dodecanoate | C_14_H_28_O_2_ | 106-33-2 | Alfa Aesar |
| 42 | Benzyl benzoate | C_14_H_12_O_2_ | 120-51-4 | SIGMA-ALDRICH |
| 43 | Ethyl myristate | C_16_H_32_O_2_ | 124-06-1 | ALDRICH |
| 44 | Ethyl heptanoate | C_9_H_18_O_2_ | 106-30-9 | ALDRICH |
| 45 | 3-Methyl-1-butanol | C_5_H_12_O | 123-51-3 | SIGMA-ALDRICH |
| 46 | Geranyl acetate | C_12_H_20_O_2_ | 105-87-3 | ALDRICH |
| 47 | Nerolidol | C_15_H_26_O | 7212-44-4 | ALDRICH |
| 48 | Ethyl caprylate | C_10_H_20_O_2_ | 106-32-1 | ALDRICH |
| 49 | Ethyl caprate | C_12_H_24_O_2_ | 110-38-3 | ALDRICH |
| 50 | Bis(2-ethylhexyl) phthalate | C_24_H_38_O_4_ | 117-81-7 | SIGMA-ALDRICH |
| 51 | Dibutyl phthalate | C_16_H_22_O_4_ | 84-74-2 | Fluka |
| 52 | Butyric acid | C_4_H_8_O_2_ | 107-92-6 | ALDRICH |
| 53 | 6-Methyl-5-hepten-2-one | C_8_H_14_O | 110-93-0 | ALDRICH |
| 54 | 2,2,6-Trimethylcyclohexanone | C_9_H_16_O | 2408-37-9 | Alfa Aesar |
| 55 | Guaiacol | C_7_H_8_O_2_ | 90-05-1 | ACROS ORGANICS |
| 56 | Pentadecane | C_15_H_32_ | 629-62-9 | ALDRICH |
| 57 | 1-Undecene | C_11_H_22_ | 821-95-4 | ALDRICH |
| 58 | Benzaldehyde | C_7_H_6_O | 100-52-7 | SIGMA-ALDRICH |
| 59 | 2-Butyl-1-octanol | C_12_H_26_O | 3913-02-8 | ALDRICH |
| 60 | 3-Octanol | C_8_H_18_O | 20296-29-1 | TCI |
| 61 | Nonanoic acid | C_9_H_18_O2 | 112-05-0 | SIGMA |
