# Supplementary material for: Odor-Induced Vomiting Is Combinatorially Triggered by Palp Olfactory Receptor Neurons That Project to the Lobus Glomerulatus in Locust Brain
Source: Front Physiol. 2022 Apr 20;13:855522. doi: 10.3389/fphys.2022.855522 (PMC9065551; doi:10.3389/fphys.2022.855522)
Supplement: Supplementary file 2 [file Image1.pdf]

Figure S1

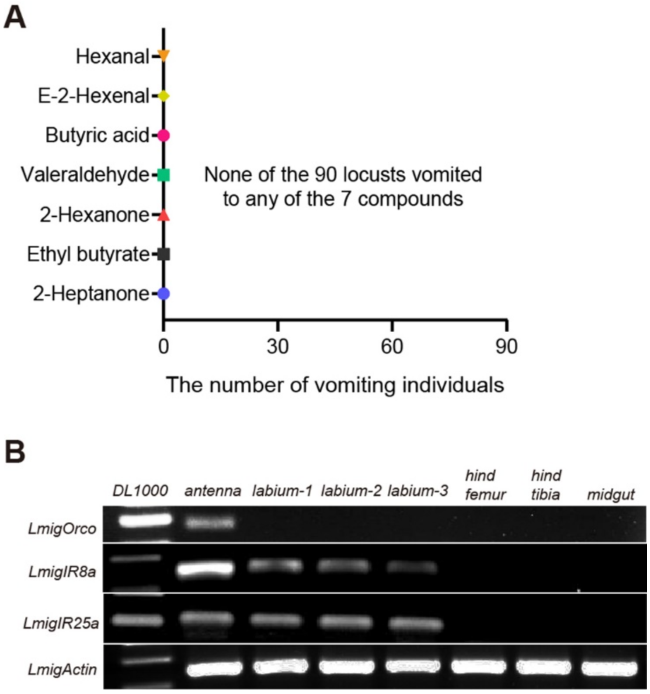

Figure S1 (A) Vomiting response assays to seven chemicals in palp-ablated locusts (antennae were intact). All chemicals were tested at 50% (v/v) dilution. *n=90* locusts per odor. **(B)** Expression of Orco, IR8a and IR25a genes in different tissues of *L. migratoria* by semi-quantitative RT-PCR. The antenna was used as a positive control, while the hind femur, hind tibia and midgut were used as negative controls. Labium-1, labium-2 and labium-3 represented three indepentent replications.

Figure S2

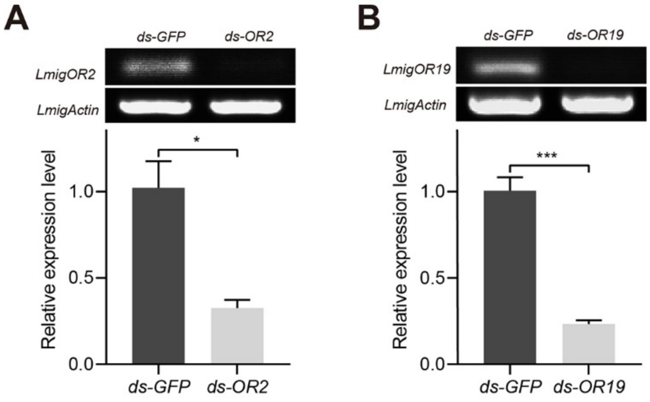

Figure S2 Effects of RNAi against OR2 **(A)** and OR19 **(B)**, *n=3*. Statistical test: unpaired two-tailed t test. Statistical differences are represented as follows: * p<0.05, *** p<0.001. Data are represented as the mean ± SEM.

Figure S3

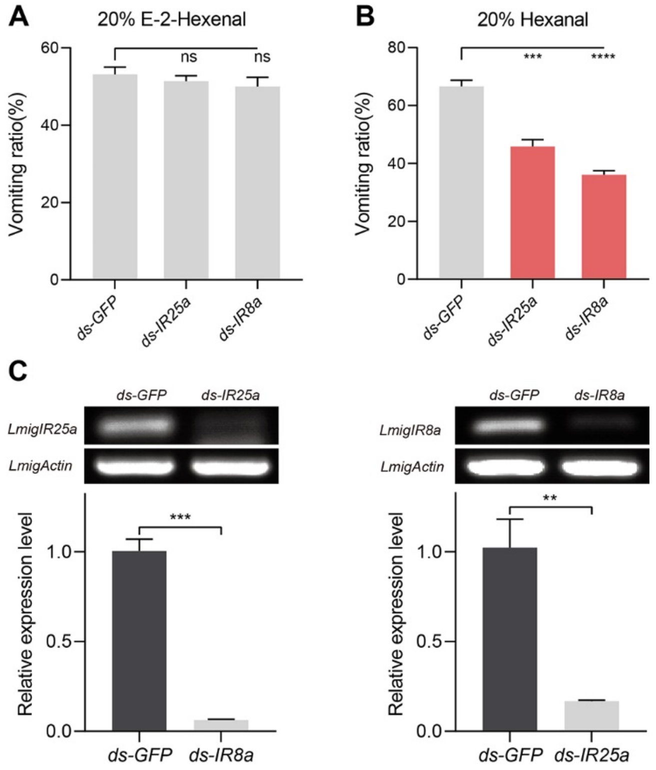

Figure S3 Ionotropic receptor pathways regulate locust vomiting induced by Hexanal. **(A,B)** Vomiting response assays toward 20% v/v E-2-Hexenal **(A)** and Hexanal **(B)** with locusts treated with dsRNA of GFP, IR25a or IR8a. Statistical test: ordinary one-way ANOVA with uncorrected Fisher's LSD test. **(C)** Effects of RNAi against IR25a and IR8a, *n=3*. Statistical test: unpaired two-tailed t test. For all analyses, statistical differences are represented as follows: ns, not significant, ** p<0.01, *** p<0.001, **** p<0.0001. Data are represented as the mean ± SEM.

Figure S4

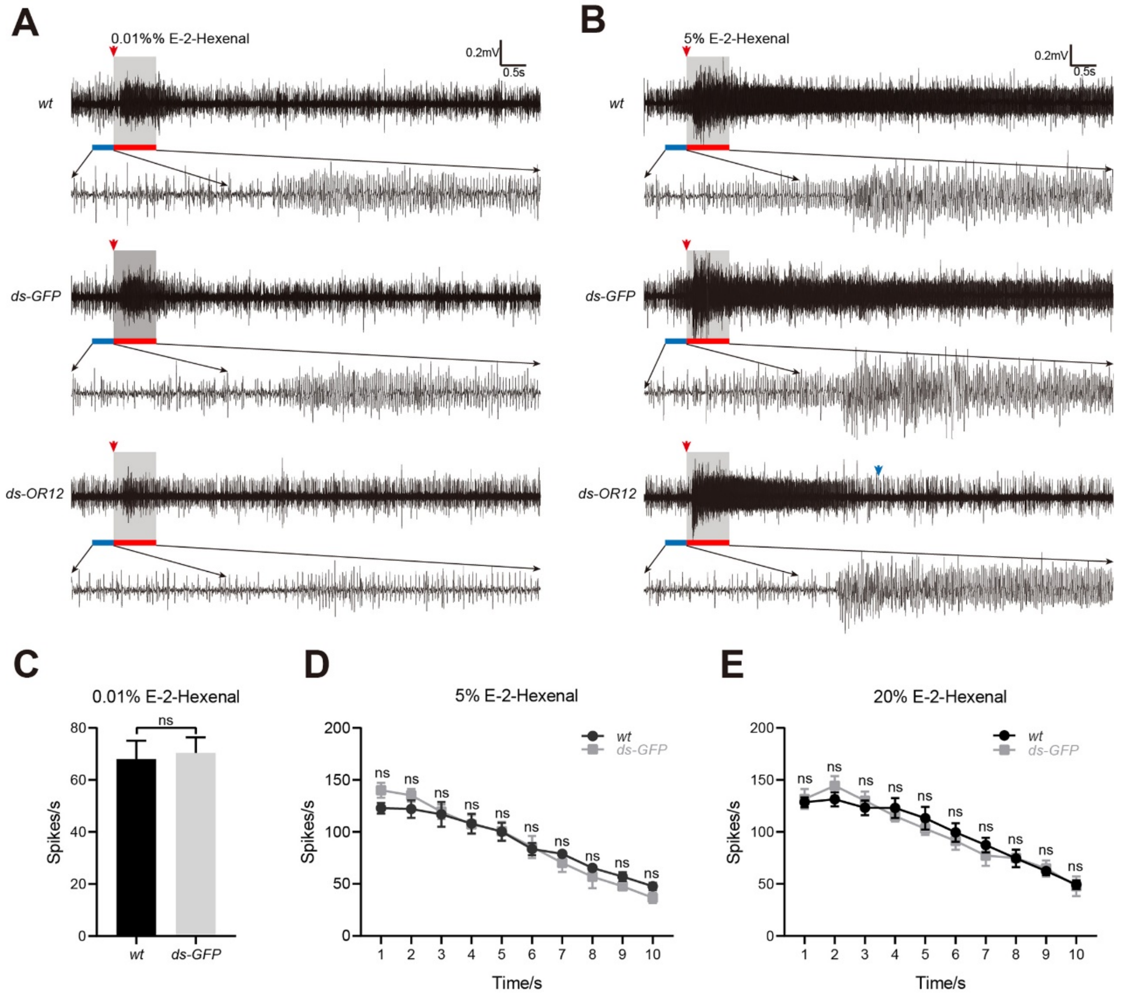

Figure S4 More electrophysiological responses to E-2-Hexenal from pb3 ORNs. **(A,B)** Representative traces showing the response of pb3 to 0.01% **(A)** and 5% **(B)** v/v E-2-Hexenal in wild-type locusts and locusts treated with dsRNA of GFP or OR12. The blue bar marks 0.5 s spontaneous activity before stimulation. Red bar marks stimulus delivery and duration (1 s). The red arrow indicates the point at which the stimulus begins. The blue arrow indicates the point at which the reaction ends. **(C)** Quantification of mean changes of all spikes in 1 s before and after 0.01% (v/v) E-2-hexenal stimulus. *n=6-10* sensilla. Statistical test: unpaired two-tailed t test. **(D,E)** Quantification of mean changes of spikes per 1 s within 10 s after 5% **(D)** and 20% **(E)** v/v E-2-hexenal stimulus, △spikes= the number of spikes per 1 s within 10 s after stimulation - the number of spontaneous spikes in 1 s before stimulation. *n=6-10* sensilla. Statistical test: two-way ANOVA with uncorrected Fisher's LSD test. For all analyses, statistical differences are represented as follows: ns, not significant. Data are represented as mean ± SEM.

Figure S5

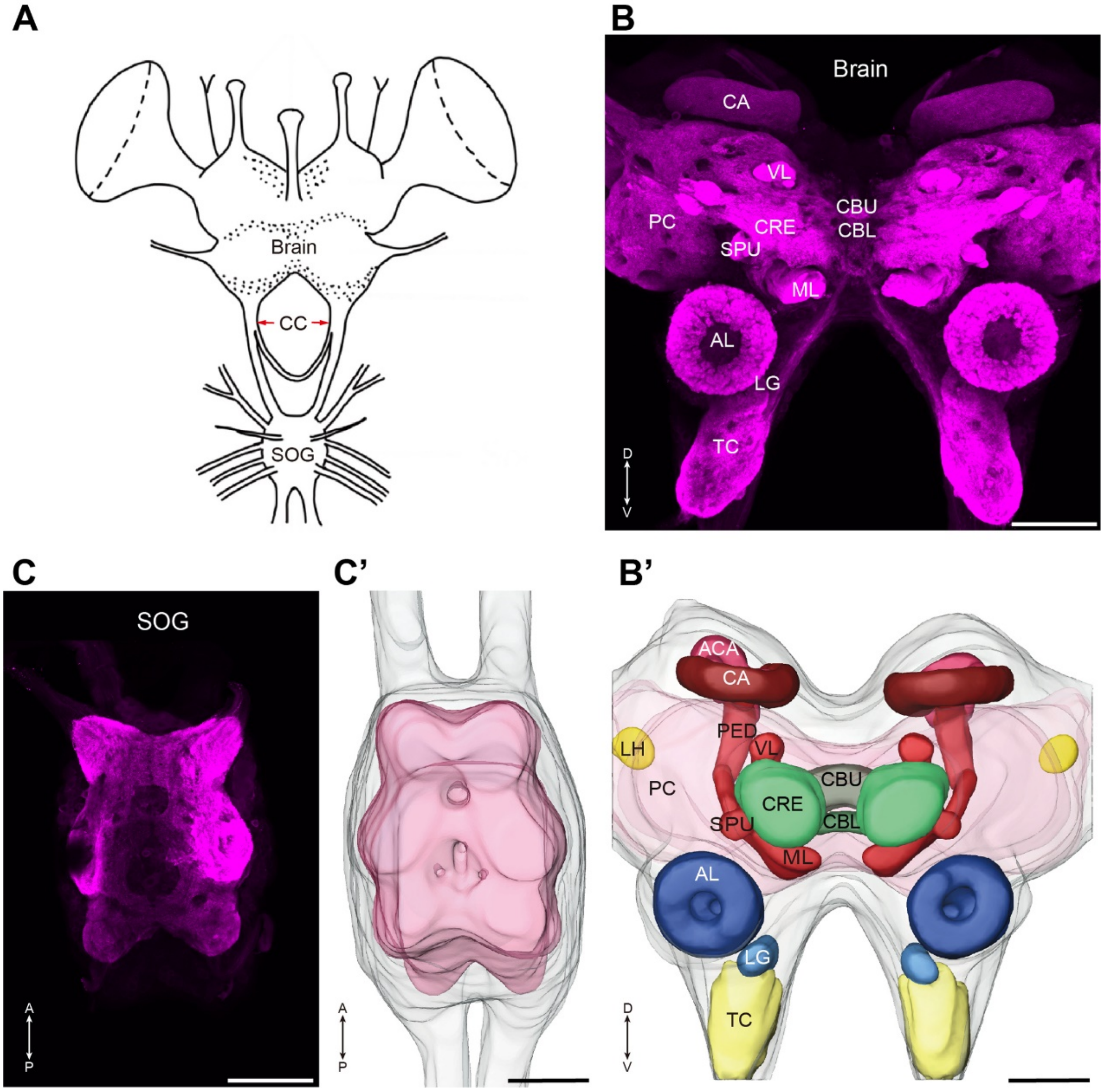

Figure S5 Registration of the brain and SOG of 5th instar nymphs. **(A)** Schematic diagram of the locust central nervous system, including the brain and the SOG. **(B,C)** Confocal image of the brain **(B)** and SOG **(C)** stained with anti-synapsin. **(B',C')** Three-dimensional reconstruction of the brain **(B')** and the SOG **(C')** shown in **(B)** and **(C)**. CC circumoesophageal connective, SOG suboesophageal ganglion, TC tritocerebrum, LG lobus glomerulatus, AL antennal lobe, PC protocerebrum, ML medial lobe, VL vertical lobe, SPU spur, CRE crepine, PED pedunculus, CA calyx, ACA accessory calyx, CBU upper division of the central body, CBL lower division of the central body, LH lateral horn, A anterior, D dorsal, P posterior, V ventral. Scale bars: 200 µm.

Figure S6

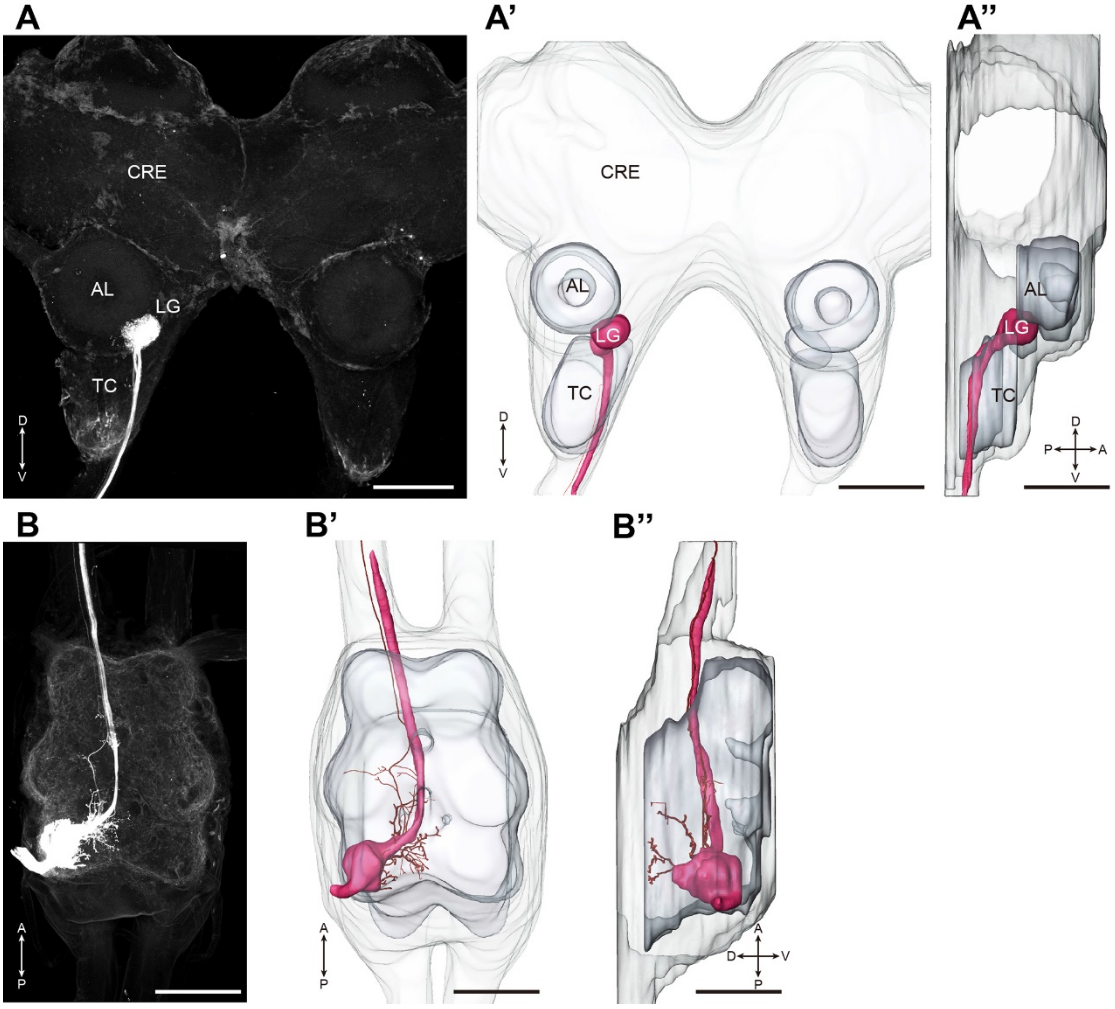

Figure S6 Central projection pattern of the sensory neurons housed in the labial palp. **(A,B)** Confocal images of the brain **(A)** and the SOG **(B)** with stained afferents originating from the maxillary palp. **(A',A'')** Three-dimensional reconstruction of the stained neurons shown in **(A)** (frontal and lateral view, respectively). **(B',B'')** Three-dimensional reconstruction of the stained neurons shown in **(B)** (frontal and lateral view, respectively). SOG suboesophageal ganglion, TC tritocerebrum, LG lobus glomerulatus, AL antennal lobe, CRE crepine, A anterior, D dorsal, P posterior, V ventral. Scale bars: 200 µm.
